# Preconditioned Chorionic Villus Mesenchymal Stem/Stromal Cells (CVMSCs) Minimize the Invasive Phenotypes of Breast Cancer Cell Line MDA231 In Vitro

**DOI:** 10.3390/ijms24119569

**Published:** 2023-05-31

**Authors:** Abdullah Al Subayyil, Yasser S. Basmaeil, Hayaa Bin Kulayb, Maha Alrodayyan, Lama Abdulaziz A. Alhaber, Taghreed N. Almanaa, Tanvir Khatlani

**Affiliations:** 1 Blood and Cancer Research Department, King Abdullah International Medical Research Center (KAIMRC), King Saud Bin Abdulaziz University for Health Sciences (KSAU), Ministry of National Guard Health Affairs (MNGHA), Riyadh 11426, Saudi Arabia; 2 Department of Botany and Microbiology, College of Science, King Saud University, Riyadh 11451, Saudi Arabia

**Keywords:** chorionic villus MSCs, MDA231, HMECs, conditioned media, adhesion, proliferation, migration, invasion, flow cytometry, epithelial to mesenchymal transition (EMT)

## Abstract

Among the newer choices of targeted therapies against cancer, stem cell therapy is gaining importance because of their antitumor properties. Stem cells suppress growth, metastasis, and angiogenesis, and induce apoptosis in cancer cells. In this study, we have examined the impact of the cellular component and the secretome of preconditioned and naïve placenta-derived Chorionic Villus Mesenchymal Stem Cells (CVMSCs) on the functional characteristics of the Human Breast Cancer cell line MDA231. MDA231 cells were treated with preconditioned CVMSCs and their conditioned media (CM), followed by an evaluation of their functional activities and modulation in gene and protein expression. Human Mammary Epithelial Cells (HMECs) were used as a control. CM obtained from the preconditioned CVMSCs significantly altered the proliferation of MDA231 cells, yet no change in other phenotypes, such as adhesion, migration, and invasion, were observed at various concentrations and time points tested. However, the cellular component of preconditioned CVMSCs significantly inhibited several phenotypes of MDA231 cells, including proliferation, migration, and invasion. CVMSCs-treated MDA231 cells exhibited modulation in the expression of various genes involved in apoptosis, oncogenesis, and Epithelial to Mesenchymal Transition (EMT), explaining the changes in the invasive behavior of MDA231 cells. These studies reveal that preconditioned CVMSCs may make useful candidate in a stem cell-based therapy against cancer.

## 1. Introduction

Although tremendous advances have been made in the early detection, prevention, diagnosis, and treatment of cancer, it remains the major cause of overall deaths worldwide [1]. Among all the cancers, breast cancer has replaced lung cancer as the most diagnosed cancer globally [2,3]. It accounts for one out of eight new cancer diagnoses. In 2020 alone, it was the most diagnosed cancers in women, and an estimated 685,000 patients, corresponding to 16% of women, died from breast cancer [1]. Although great improvements have been made in cancer therapies, including targeted therapies, biomarker driven treatment approaches, and combinatorial therapies, that have resulted in extending the overall survival of cancer patients, yet the success in the fight against cancer remains disappointing to a large extent [4]. The specificity and heterogeneity of tumors remain the main obstacle for the successful outcome of traditional therapies including chemotherapy, radiotherapy, and resection by surgery [2,3]. To overcome the drawbacks of the conventional therapies, new technologies have been proposed, developed, and tested in cancer patients. They include cancer vaccines, monoclonal antibodies, and cell-based therapies such as chimeric antigen receptor (CAR)-T-cell therapy and stem cell-based therapies [4]. In spite of their diversity and heterogeneity, and still being in the early phases of development [5], stem cells are gaining traction as a popular choice of treatment against cancer because of their antitumor effects, specific target effects through homing, and minimal off-target effects [6,7].

Stem cells isolated from adult tissues, Mesenchymal Stem/Stromal Cells (MSCs), exhibit enormous potential to be used in stem cell-based therapies. They are isolated from different tissues such as bone marrow, umbilical cord, dental pulp, adipose tissue, and from human term placenta [8]. MSCs have the capacity to grow on plastic, exhibit self-renewal capabilities, and differentiation potential. Upon stimulation, they differentiate into adipocytes, chondrocytes, and astrocytes [9]. Due to their varied features, such as differentiation capacity, homing to the injured and inflamed sites, the modulation of immune responses, and pro-proliferative and migratory potential, MSCs are considered as attractive candidates in immunomodulatory disorders, regenerative therapy approaches, and as therapeutic agents against cancer [10]. Furthermore, like wound and tumor microenvironment share similarities, MSCs have shown to respond in a similar way to cancer associated inflammatory signals and home to the tumor microenvironment [11,12].

Various in vitro studies have reported the antitumor and pro-apoptotic properties of MSCs through the inhibition of tumor growth and metastasis, modulating immune responses, impeding angiogenesis, regulating the cell cycle, and the induction of apoptosis [13]. In co-culture experiments, umbilical cord-derived stem cells have shown apoptosis of GBM cell lines mediated through the tumor necrosis factor (TNF)-related apoptosis-inducing ligand (TRAIL) [14]. Multiple animal studies have also confirmed the antitumor effects of naive MSCs and demonstrated that MSC therapy decreases the growth of glioma, melanoma, and lung and breast cancers [15]. In a melanoma mouse model, the subcutaneous injection of MSCs-initiated apoptosis resulted in tumor regression [16]. In addition, preconditioned MSCs have been shown to secrete various bioactive molecules in their conditioned media, with pro-apoptotic and anti-proliferative properties, making them a promising choice for targeted therapies against cancer [17,18].

We have previously reported the isolation and characterization of Decidua Basalis Mesenchymal Stem Cells (DBMSCs), Decidua Parietalis Mesenchymal Stem Cells (DPMSCs), and Chorionic Villus Mesenchymal Stem Cells (CVMSCs) from human term placenta and have studied their immunomodulatory properties [19,20,21,22]. We have recently reported that, after preconditioning, CVMSCs retain the ability to survive, adhere, and migrate in a medium that mimics the cancer microenvironment [23]. Preconditioned CVMSCs exhibited an increased expression of genes with anti-cancer properties, demonstrating that, in addition to retaining their normal function, they also express anti-tumor molecules in the tumor setting [23].

For clinical applications of CVMSCs in cell-based therapies against tumors, it is pertinent to evaluate the physiological effect of the CVMSCs as cells, and their secretome in the form of conditioned media on the tumor cell lines. In this study, we have investigated the effects of the cellular component and the secretome of CVMSCs on the functional consequences of the human breast cancer cell line MDA-MB-231 (MDA231). After performing a spatial and temporal treatment of MDA231 cells with the CVMSCs and their conditioned media (CM), we evaluated their functional and phenotypic properties such as adhesion, proliferation, migration, and invasion. The genomic analysis of various prominent genes that play important roles in breast cancer development, progression and Epithelial to Mesenchymal Transition (EMT) was evaluated by mRNA analysis and verified by flow cytometry.

## 2. Results

### 2.1. The Standardization of CM Concentration, Cellular Ratios, and Preconditioning Time

We evaluated the optimum concentration of CM from preconditioned CVMSCs, as well as the appropriate number of CVMSCs which had a quantifiable impact on the functional outcome of the MDA231 cells. We selected the CM concentration dose at 5%, 10%, and 25%, and subsequently four cellular ratios of 2:1, 1:1; 1:2, and 1:5 for MDA231 to CVMSCs and HMECs to CVMSCs cells were used in all experiments. For cellular contact, the MDA231 or HMECs were treated in intracellular (IC) settings. The MDA231 cells were incubated for 24 H, 48 H, and 72 H, after the CM or cellular component treatment of CVMSCs. The MTS assay was performed on treated MDA231 cells to assess their temporal effects.

**Figure 1:**
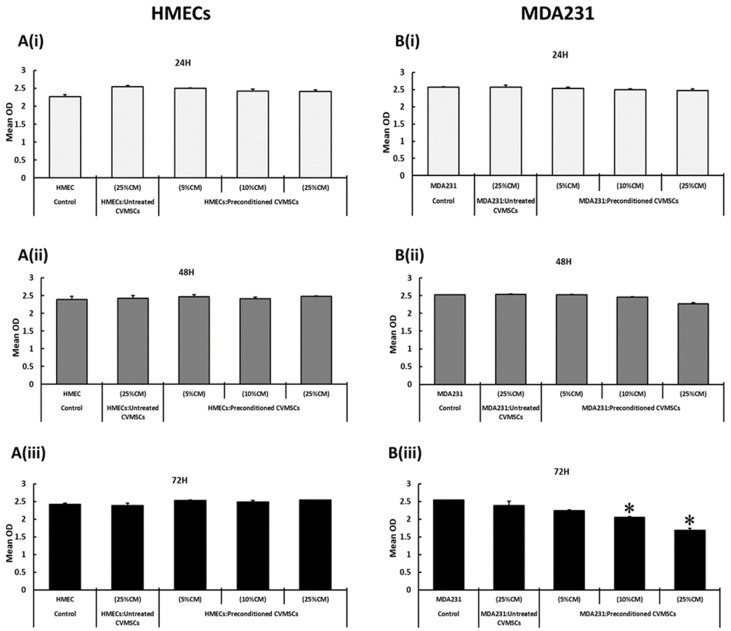
Standardization of incubation time and CM dose for treatment of MDA231 and HMEC cells: the effect on HMEC and MDA231 cells in response to different concentrations of CM-CVMSCs at 24 H, 48 H, and 72 H post treatment, using MTS assay. The CM of preconditioned CVMSCs did not change the proliferation of HMEC cell proliferation at any of the CM concentrations tested at any time point tested (**A**(**i–iii**)). Although, after treatment with CM of preconditioned CVMSCs for 24 and 48 H (**B**(**i**,**ii**)), the proliferation of MDA231 cells did not change in response to the CM concentration of 5% and 10%, at 25% ratio their proliferation reduced significantly at 72 H treatment (**B**(**iii**)) as compared to untreated controls. Each experiment was repeated three times with CM collected from preconditioned and naïve CVMSCs isolated from five different placentas. Bars represent standard errors. * *p* < 0.05.

To determine the appropriate dose of CM-CVMSCs, which has a measurable effect on the functional characteristics of MDA231 cells, three doses of CM-CVMSCs at 5%, 10%, and 25% (CM obtained from preconditioned CVMSCs diluted in complete medium to obtain the working concentration doses) were selected to treat the HMEC and MDA231 cells. After treatment, the cells were incubated at 24 H, 48 H, and 72 H, which was followed by the MTS assay. Figure 1A(i–iii) shows that the CM of preconditioned CVMSCs did not modulate the proliferation of HMEC cells at any of the doses tested at any of the time points selected, although in the MDA231 cells as shown in Figure 1B(i,ii), after 24 H and 48 H of treatment, no concentration tested modulated the proliferation of MDA231 cells in the MTS assay. However, after 72 H (Figure 1B(iii)) of sustained treatment with CM of CVMSC, the MDA231 cells showed a dose-dependent reduction in overall proliferation, which reached to the significant level (*p* < 0.05) at 10% and 25% CM of CVMSCs against 5% and CM of naïve CVMSCs used as control. Since this response was more robust and significant (*p* < 0.05) at 25% of treatment after incubation for 72 H, the exposure time of 72 H was chosen for the treatment of MDA231 cells with 25% of CM and was selected to study their effect on the functional outcome of MDA231 cells.

**Figure 2:**
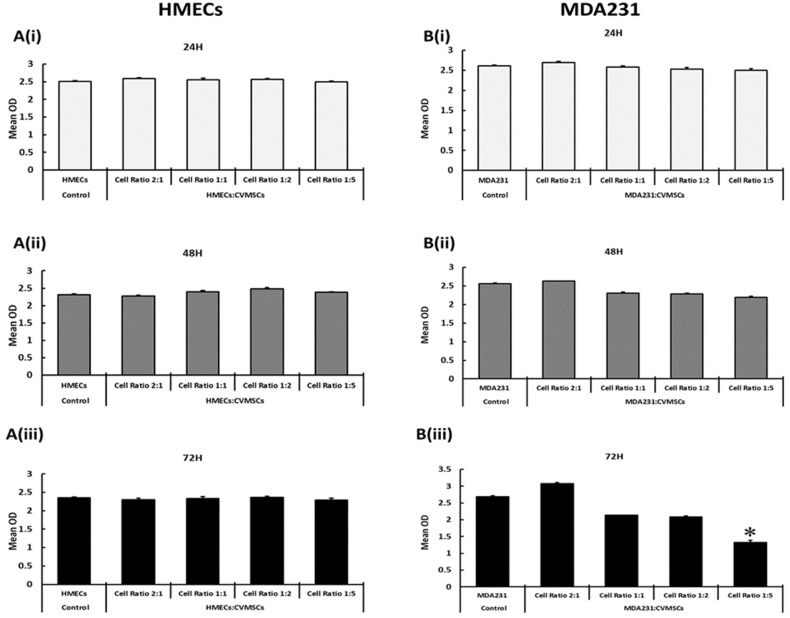
Standardization of co-culture time and cellular ratio for treatment of MDA231 and HMEC cells: the cellular component of preconditioned CVMSCs did not have any measurable effect on HMEC cell proliferation at various time points (24 H, 48 H and 72 H) and at various cellular ratios tested (**A**(**i**–**iii**)). However, after treatment with preconditioned CVMSCs for 24 H and 48 H (**B**(**i**,**ii**)), the proliferation of MDA231 cells did not change in response to the cellular ratios of 2:1, 1:1, 1:2, or 1:5 pertaining to MDA231 to CVMSCs cells, and as compared to untreated controls. In comparison, at 72 H post treatment, MDA231 cell proliferation decreased in a dose-dependent manner at the cellular ratios of 1:1 and 1:2, but at the 1:5 ratio (between the MDA231 and CVMSCs) their proliferation reduced significantly as compared to the untreated control cells (**B**(**iii**)). Each experiment was repeated three times with preconditioned and naïve CVMSCs isolated from five different placentas. Bars represent standard errors. * *p* < 0.05.

HMEC cells co-cultured with preconditioned CVMSCs in the IC setting did not show any modulation in proliferation at 2:1, 1:1; 1:2, or 1:5 cellular ratio (HMECs: CVMSCs) at any of the time points tested, as shown in Figure 2A(i–iii). Although MDA231 cells also did not exhibit any change in proliferation after treatment with CVMSCs at 24 and 48 H at 2:1, 1:1; 1:2, or at 1:5 (Figure 2B(i,ii)) cellular ratio (MDA231: CVMSCs), at a cellular ratio of 1:5 and after 72 H of incubation, MDA231 cells showed significantly reduced proliferation (*p* < 0.05), as shown in Figure 2B(iii), as compared to the untreated control. Thus, an exposure time of 72 H was chosen for the treatment of MDA231 cells with CVMSCs at 1:5 cellular ratio to study their functional consequences.

### 2.2. CM-CVMSCs Enhance Proliferation but No Other Phenotype of MDA231 Cells

MDA231 cells were incubated with CM of preconditioned CVMSCs for 72 H at a ratio of 1:5. The cells were washed with PBS, before harvesting and being subjected to cell analysis by xCELLigence Real-Time Cell Analyzer (RTCA) to measure the effect of CM on MDA231 cells. Untreated MDA231 cells in complete medium and the cells treated with CM of naïve CVMSCs served as control. The cell viability of MDA231 cells after treatment was evaluated by Trypan Blue and was found at >95%.

**Figure 3:**
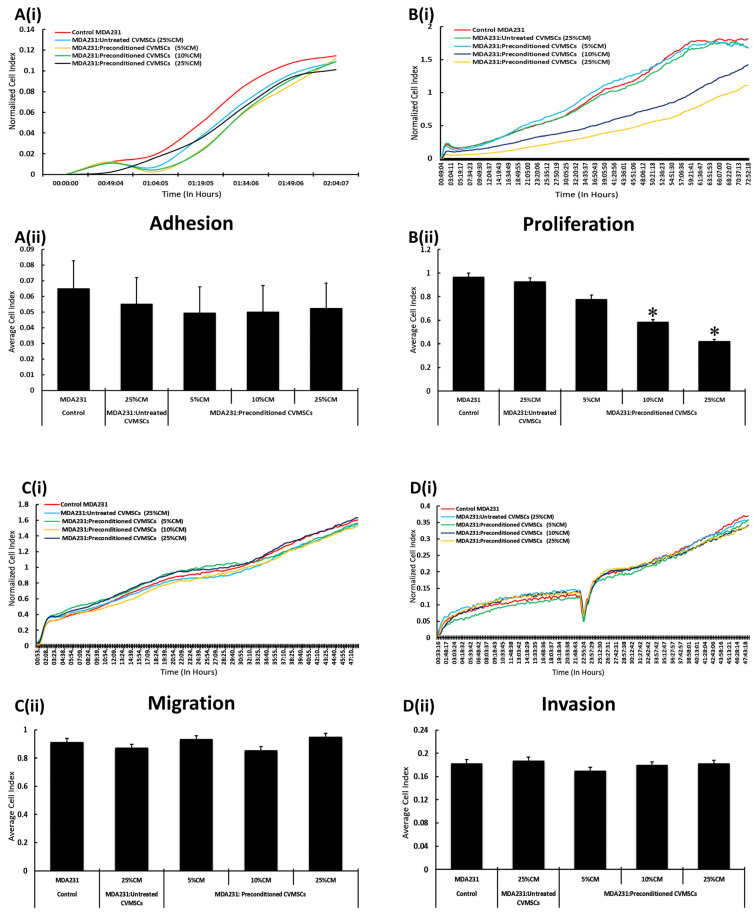
Effect of CM from preconditioned CVMSCs on MDA231 functions: MDA231 functions including cellular adhesion, proliferation, migration, and invasion, were evaluated with the xCELLigence RTCA system. In response to different concentrations of CM from preconditioned or naïve CVMSCs, MDA231 cell adhesion decreased as compared to untreated control, yet the effect was not statistically significant (**A**(**i**,**ii**)). However, proliferation of MDA231 cells decreased significantly after treatment with 10% and 25% CM as compared to the 5% CM treatment and to the untreated control (**B**(**i**,**ii**)). MDA231 cellular migration and invasion did not change significantly when treated with the CM of preconditioned CVMSCs at 5%, 10%, or at 25% concentrations, as compared to the untreated control (**C**(**i**,**ii**),**D**(**i**,**ii**)). Each experiment was repeated three times with CM collected from preconditioned CVMSCs isolated from five different placentas. Bars represent standard errors. * *p* < 0.05.

As shown in Figure 3A(i,ii), the adhesion of MDA231 to the extracellular matrix did not change when treated with CM of preconditioned CVMSCs at 5%, 20%, or 25% as compared to the 25% CM from naïve CVMSCs and untreated control MDA231 cells. Figure 3A(i) shows the normalized cell index during the first two hours of the experiment, whereas Figure 3A(ii) depicts the bar diagram of the average cell index, recorded in the first two hours of the experiment.

Treatment with 10% and 25% of CM from preconditioned CVMSC and incubated for 72 H reduced the proliferation of MDA231 significantly (*p* < 0.05) as compared to the treatment with 25% CM from naïve CVMSCs and untreated control MDA231 control cells. Figure 3B(i) shows the normalized cell index recorded during 72 H of the experiment, whereas Figure 3B(ii) depicts the mean cell index in the bar diagram, comparing different treated groups with the 25% CM from naïve CVMSCs-treated MDA231 cells and the untreated control cells.

Contrary to increased proliferation, and as compared to the untreated control and after treatment with CM from the untreated CVMSCs, the MDA231 cells treated with CM from preconditioned CVMSCs at 5%, 10% and 25% concentration did not depict any change in cellular migration as evaluated in the xCELLigence RTCA system (Figure 3C(i)). The average cell index calculated from the xCELLigence RTCA data reflected a similar trend, with no significant change in the cellular migration of MDA231 cells against the control groups (Figure 3C(ii)).

Like migration, the cellular invasion of MDA231 cells incubated with CM of preconditioned CVMSCs at 5%, 10%, or 25% did not show any significant reduction as compared to CM from naïve CVMSCs, either treated or the untreated control (Figure 3D(i)). The average cell index of the invading cells reflected the trend, calculated from the data obtained in the xCELLigence RTCA analysis (Figure 3D(ii)).

### 2.3. Preconditioned CVMSCs Suppress the Invasive Phenotype of MDA231 Cells

**Figure 4:**
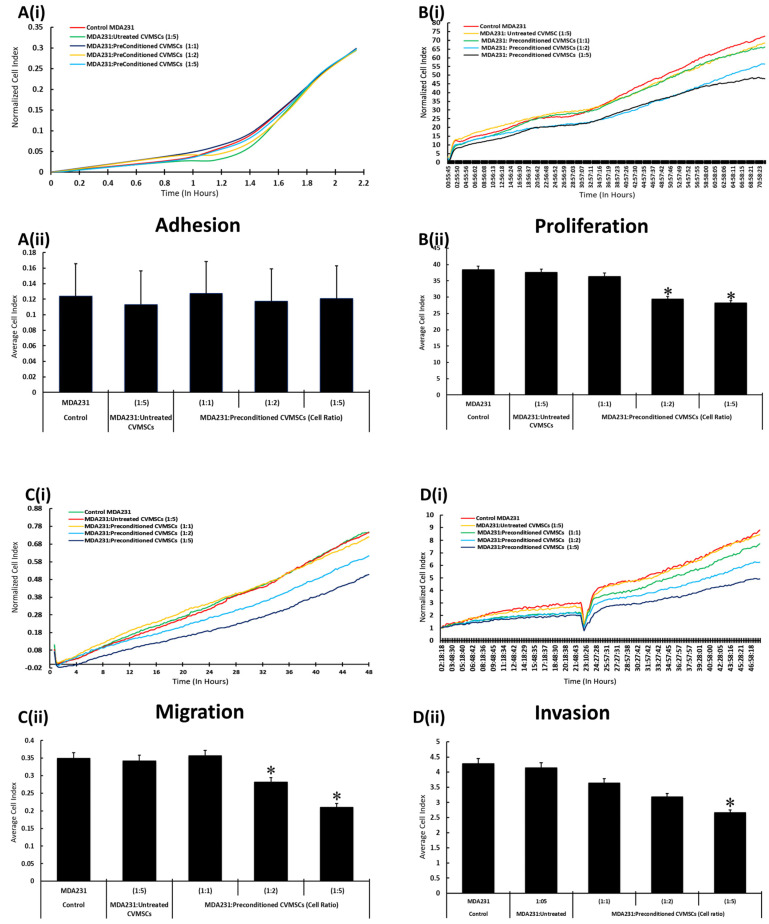
Effect of the cellular component of preconditioned CVMSCs on MDA231 functions: after treatment with preconditioned or with naïve CVMSCs in the IC setting, MDA231 functions such as cellular adhesion, proliferation, migration, and invasion, were evaluated by the xCELLigence RTCA system. In response to different cellular ratios (1:1; 1:2, and 1:5) of preconditioned or naïve CVMSCs, MDA231 cell adhesion did not change, as compared to the untreated control MDA231 cells (**A**(**i**,**ii**)). However, proliferation of MDA231 cells decreased significantly after treatment with preconditioned CVMSCs at 1:2 and 1:5 cellular ratios (MDA231: CVMSCs) and as compared to the 1:1 cellular ratio and to the untreated control (**B**(**i**,**ii**)). MDA231 cell migration decreased significantly when treated with the cellular component of preconditioned CVMSCs at both 1:2 and 1:5 cellular ratios in IC setting, as compared to the untreated control (**C**(**i,ii**)). Invasion of MDA231 changed significantly when treated with preconditioned CVMSCs at 1:2 and 1:5 cellular ratios in IC setting. However, at 1:1 ratio, the invasiveness of MDA231 cells did not change significantly as compared to the untreated control cells (**D**(**i**,**ii**)). Each experiment was repeated in triplicate with CVMSCs isolated from five different placentas. Bars represent standard errors. * *p* < 0.05.

MDA231 cells co-cultured with preconditioned CVMSCs at the cellular ratios of 1:1, 1:2, and 1:5, pertaining to MDA231 cells to CVMSCs, did not show any significant change in their adhesion potential as compared to MDA231 cells co-cultured with naïve cells or the untreated control (Figure 4A(i)). The average cell index of the adhesion data as recorded in the xCELLigence RTCA analysis shows the similar trend, and is recorded as a bar diagram, as shown in (Figure 4A(ii)).

MDA231 cells exhibited a significant decrease in proliferation (*p* < 0.05) after treatment with preconditioned CVMSCs at 1:2 and 1:5 cellular ratios (MDA231: CVMSCs), as compared to treatment with naïve CVMSCs at 1:5 cellular ratios and in untreated controls in xCELLigence RTCA system (Figure 4B(i)). As shown in Figure 4B(ii), the growth curves obtained in the xCELLigence RTCA analysis corresponded to the average cell indices calculated for each experimental group, whereas MDA231 cells showed a significant reduction in proliferation in co-culture settings pertaining to 1:2 and 1:5 (MDA231: CVMSCs) cellular ratios.

In comparison to the results obtained for MDA231 cells after treatment with the CM of preconditioned CVMSCs for migration potential, the co-culture of MDA231 cells with preconditioned CVMSCs depicted decreased at both 1:2 and 1:5 (MDA231: CVMSCs) cellular ratios compared to the cells treated with naïve CVMSCs and the untreated control, as shown in Figure 4C(i)). Although a reduction in MDA231 cellular migration was observed at both 1:2 and 1:5 (MDA231: CVMSCs) co-culture settings, the decrease at 1:5 (MDA231: CVMSCs) was found to be statistically significant (Figure 4C(ii). Similar results were obtained for MDA231 cellular invasion when they were co-cultured with preconditioned CVMSCs at 1:2 and 1:5 cellular ratios as compared to treatment with untreated CVMSCs and to the untreated control MDA231 cells (Figure 4D(i)). As observed for the migratory potential after co-culture, the MDA231 cells showed a significant decrease (*p* < 0.05) in cellular invasion at the cellular ratio of 1:5 (MDA231: CVMSCs) against the control groups, as shown in Figure 4D(ii).

**Figure 5:**
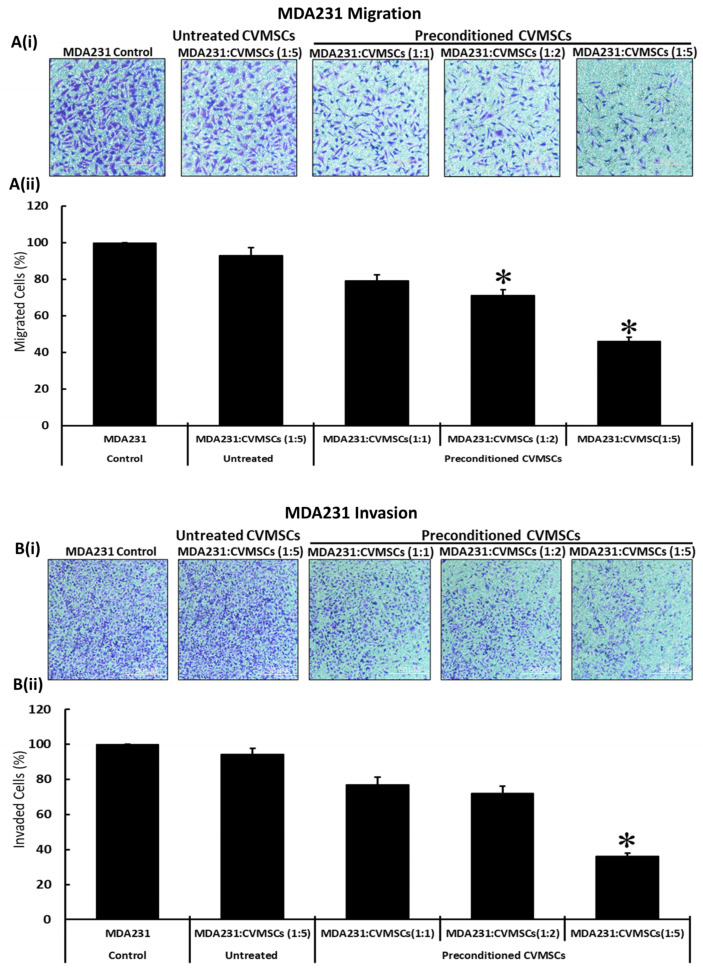
Effect of cellular component of preconditioned CVMSCs on MDA231 functions: migration of MDA231 cells after treatment with preconditioned CVMSCs in IC settings for 72 H was evaluated by transwell cell migration assay. Treated MDA231 cells with preconditioned CVMSCs in IC (at 1:2 and 1:5 cellular ratios) migrated at a significantly slower rate through the 8 µM pore of a transwell filter as compared to 1:1 cellular ratios and untreated control cells. Panel (**A**(**i**)) shows the photomicrographs of the migrated cells under various treatment conditions. After staining, the migrated cells in five fields were counted and their percentage is presented in a bar graph (**A**(**ii**)). Invasion of MDA231 cells treated with preconditioned CVMSCs in IC setting was examined by a Matrigel coated transwell filter with a pore size of 8 µM, as described in the Material and Methods section. MDA231 showed a significant reduction in cellular invasion at 1:5 cellular ratio (MDA231: CVMSCs) in the IC setting as compared to the 1:1 and 1:2 cellular ratios and untreated control experiment groups. Panel (**B**(**i**)) shows the photomicrographs of MDA231 cells that invaded through the Matrigel coated membrane cells under different treatment conditions. The invaded cells were stained and counted in five different fields. The percentage of invaded cells is presented as a bar graph (**B**(**ii**)). Each experiment was repeated in triplicate with CVMSCs isolated from five different placentas. Scale bars, 100 μM. Values are represented as means ± SE, * *p* < 0.05.

To confirm the modulation in migratory and invasive phenotypes of MDA231 cells after co-culture with preconditioned CVMSCs, a transwell assay was performed to corroborate the data obtained by the xCELLigence RTCA system. After co-culture for 72 H in IC settings at 1:1; 1:2, and 1:5 ratios (MDA231: CVMSCs), the cells were made to pass through an insert with a pore size of 8 µM for migration assays. As shown in Figure 5A(i,ii), and as compared to the untreated control and the cells treated with naïve CVMSCs, the co-culture of MDA231 cells with preconditioned CVMSCs at 1:1; 1:2, and 1:5 cellular ratios exhibited a decrease in number of cells that migrated through the membrane (Figure 5A(i)). The number of cells migrated though the membrane (shown as percent migrated cells) was significantly reduced (*p* < 0.05) for 1:2 and 1:5 co-cultured MDA231 cells as compared to the untreated control (Figure 5A(ii)).

The invasion of MDA231 cells treated with the cellular component of preconditioned CVMSCs and the naïve CVMSCs was further examined by transwell assay using inserts coated with Matrigel. The infiltration of MDA231 cells through the Matrigel-coated membrane defines their invasion potential. MDA231 cells which had invaded through the membrane were stained with crystal violet and counted. The invasion of MDA231 cells co-cultured with preconditioned CVMSCs at ratios of 1:2 and 1:5 (MDA231: CVMSCs) in the IC setting decreased as compared to co-culturing with untreated CVMSCs and untreated controls (Figure 5B(i)). The number of invaded cells though the Matrigel-coated membrane (shown as percent migrated cells) was significantly reduced (*p* < 0.05) in MDA231 cells co-cultured with preconditioned CVMSCs at a 1:5 ratio, and as compared to the untreated control cells (Figure 5B(ii)), indicating that preconditioned CVMSCs, but not the naïve CVMSC, alter the invasive potential of MDA231 cells.

These results are in agreement with the results obtained in the xCELLigence RTCA assays for both the migration and invasion of MDA231 cells after treatment with preconditioned CVMSCs.

### 2.4. CVMSCs Modulate the Expression of Genes Responsible for Breast Cancer Oncogenesis and Metastasis in MDA231 Cells

The modulation of functionally relevant effectors responsible for phenotypic changes and EMT in MDA231 cells after treatment with preconditioned CVMSCs or their secretome was evaluated by employing RT-PCR analysis. We used an RT² Profiler™ PCR Array Human Breast Cancer kit (Qiagen Cat# PAHS-131ZR) to analyze modulation in the expression of molecules involved in breast oncogenesis and cancer progression at the RNA level. Simultaneously, we used RT² Profiler™ PCR Array Human Epithelial to Mesenchymal Transition (Qiagen Cat# PAHS-090ZA) to analyze the modulation in expression of genes responsible for EMT at the RNA level. Table 1 shows the expression of several molecules with tumor suppressor or oncogenic properties in MDA231 cells after treatment with the cellular component or secretome of preconditioned CVMSCs.

RNA expression levels of various functionally important oncogenes such as *Cyclin D1, SCF1, EGFR, ERBB2, GSTP1, IDO, IL6, NOTCH1,* and *TGFB1* were downregulated in MDA231 cells treated either with 25% CM or directly with the cellular component of preconditioned CVMSCs at a 1:5 cell ratio between MDA231 and CVMSCs. However, a reduction in expression of other oncogenes including *ATM, BIRC5, Cyclin D2, Cyclin E1, ESR1, IGFBP3, MMP2, and MMP7* was found only in MDA231 cells treated with the cellular component of preconditioned CVMSCs, but not with their CM. In addition, several tumor suppressor genes including *CDKN1A, CDKN1C, IFN-γ, CDH1*, and *RB1* were upregulated in MDA231 cells after treatment with preconditioned CVMSCs in the IC setting, as well as after their treatment with the CM from preconditioned CVMSCs.

The RNA expression levels of various effector molecules often upregulated during EMT (Supplementary Table S1), such as *BMP1, COL3A1, COL5A2, FOXC2, TIMP1, VCAN, WNT5B, CALD1, CAMK2N1, CDH2, FN1, MMP9, SNAI1, SPARC*, and *TMEM132A*, were downregulated in MDA231 cells treated either with 25% CM or directly with the cellular component of preconditioned CVMSCs at a 1:5 cell ratio between MDA231 and CVMSCs. In addition, several genes including *CAV2, FGFBP1, KRT19, MST1R, OCLN*, and *RGS2* were upregulated in MDA231 cells after treatment with preconditioned CVMSCs as well as after their CM. A significant modulation in the expression of genes involved in metastasis and EMT, as observed in MDA231 cells after treatment with CM and with the cellular component of preconditioned CVMSCs, was directly involved in various essential functions such as cell growth and proliferation, cellular migration, motility and invasion, differentiation and development, and cellular adhesion (Supplementary Table S2).

**Figure 6:**
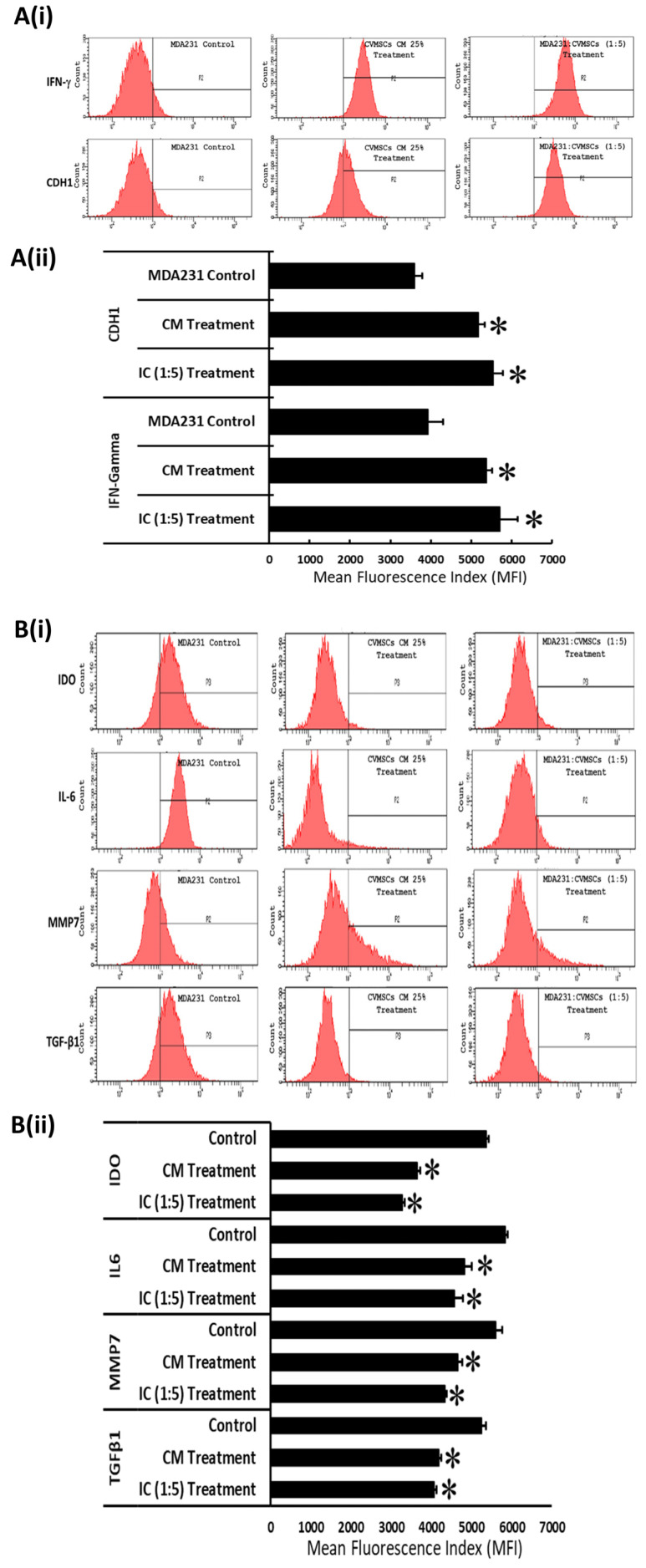
Modulation in expression of tumor suppressor proteins and oncogenes in MDA231 cells treated with preconditioned CVMSCs: flow cytometry analysis for the expression of tumor suppressor proteins modulated in MDA231 cells after treatment with preconditioned CVMSCs showed significant increase in the expression levels for CDH1 and IFN-gamma in both IC and CM settings as compared to untreated control (**A**(**i**)). Expression levels of oncogenes such as IDO, IL6, MMP7, and TGF-β1 reduced significantly in MDA231 cells in both IC and SF setting as compared to untreated control (**B**(**i**)). Data obtained by FACS analysis from three independent experiments were quantified and are shown in bar graphs as Mean Florescence Index (MFI) pertaining to CDH1 and IFN-gamma (**A**(**ii**)), and IDO, IL6, MMP7, and TGF-β1 (**B**(**ii**)), respectively. Bars represent standard errors. * *p* < 0.05.

RNA expression was further validated at the proteomic level by performing a flow cytometry analysis of a few important effector molecules involved in breast oncogenesis. As shown in Figure 6A(i), the protein expression levels of tumor suppressor proteins CDH1 and IFN-γ increased significantly (*p* < 0.05) after treatment either with CM or the cellular component of the preconditioned CVMSCs. Figure 6A(ii) shows the Mean Fluorescence Index (MFI) of the expression levels observed in the flow cytometry analysis. Similarly, protein expression levels for oncogenes such as IDO, IL6, MMP7, and TGF-β1 showed a decreased trend in MDA231 cells treated either with CM or the preconditioned CVMSCs in flow cytometry analysis (Figure 6B(i)). The analysis of the data, as shown in Figure 6B(ii), depicted the decrease in expression of these oncogenes as statistically significant (*p* < 0.05) as compared to the untreated controls, as well as their expression in MDA231 cells treated with the secretome or cellular component of the naïve CVMSCs. The results of all these molecules were recorded in MFI units.

## 3. Discussion

Despite their contradictory properties, the therapeutic potentials of MSCs in various diseases, such as immune mediated, cardiovascular, diabetes, and against cancer, are still explored in various diseases [24,25]. Because of their secretion of a variety of bioactive molecules, their paracrine activity against the surrounding cells, and their migration (homing) to the sites of inflammation and injury, they are studied for clinical applications including inflammation and injury, immune modulation, angiogenic, tissue regeneration, and pro-apoptotic effects in cancer treatments [25,26,27,28,29].

We have earlier reported the isolation and characterization of CVMSCs from the chorionic villus region of human placentas. They secrete and express novel effector molecules with an ability to modify the functional activities of their target cells, along with the immunosuppressive properties by shifting pro inflammatory macrophages M1 to anti-inflammatory M2 macrophages [20,30,31]. CVMSCs not only function normally in the harsh oxidative stress environment induced by high levels of H_2_O_2_ and glucose, but also protect the endothelial cells from their damaging effects [32,33]. In addition, CVMSCs also enhance the anti-tumor properties of NK cells in vitro [34]. Furthermore, we have recently reported that CVMSCs not only survived and functioned normally in the cancer microenvironment, but their expression of pro-apoptotic and anti-cancer molecules was also enhanced in the harsh and toxic environment, mimicking that of a tumor [23]. Such distinguishing properties make CVMSCs an attractive source of therapy to treat inflammatory diseases such as cancer.

**Figure 7:**
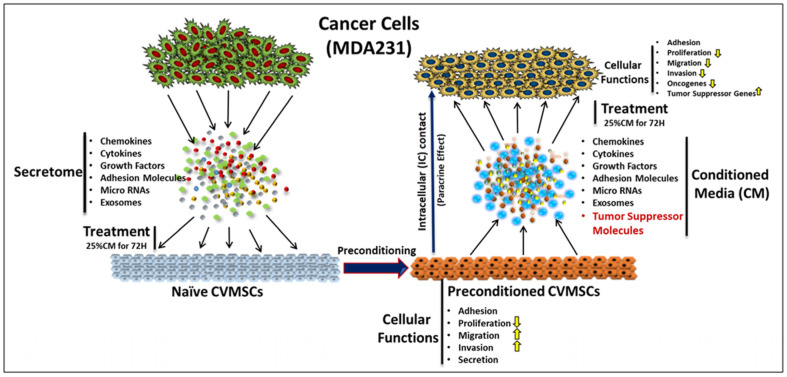
In the cancer microenvironment, tumor cells secrete a plethora of essential factors such as chemokines, cytokines, growth factors, adhesion molecules, micro-RNAs, and exosomes, etc., that exert influence on the naïve CVMSCs, educating them to survive in the tumor setting. This process, known as preconditioning, modulates the functional properties of the CVMSCs, preparing them to enhance their anti-tumor properties, in addition to other cellular functions. Preconditioned CVMSCs express and secrete a different set of molecules that contain, specifically, the tumor suppressor molecules, along with other factors such as chemokines, cytokines, growth factors, and adhesion molecules, etc. Treatment of MDA231 cells with the cellular component of preconditioned CVMSCs (paracrine effect; IC) or their secretory products (conditioned media; CM) leads to modulation of their cellular functions, including decreases in proliferation, migration, invasion, and oncogenesis. This whole process involves the decrease in expression of oncogenes and increase in the expression levels of tumor suppressor proteins.

To explore the possibility of using CVMSCs as a possible therapy against tumor, we investigated the impact of the secretome and the cellular content of CVMSCs on the breast cancer cell line MDA231. We tested the secretome and cellular content of naïve CVMSCs, as well as those from the cells incubated with the conditioned medium of MDA231 cells (preconditioning) prior to the collection of secretome (CM-CVMSCs) and the cellular content (preconditioned CVMSCs) (Figure 7 and Supplementary Figure S1).

Preconditioning, or “in vitro priming”, is a common and widely used approach to modulate the behavior of MSCs by exposing them to certain selected factors such as cytokines, interleukins, growth factors, etc., in the culture medium [35]. In vitro priming has been used to investigate the anti-inflammatory phenotype of MSCs, which could be used to enhance their migratory and homing potential [36]. The exposure of MSCs to TNF-α not only improves their adhesion to endothelial cells, but also improved their migration towards chemokines [37,38]. In addition, the exposure of MSCs to TGF-β1 resulted in enhanced migration towards glioblastoma cells [39].

To enhance the anti-tumor properties of the CVMSCs, we first preconditioned the CVMSCs using the secretome obtained from the culture of MDA231 cells. The cells were treated with different doses of the secretome at various time points, as described previously [23]. Secretome and cells preconditioned for 72 H with 25%CM of MDA231 cells were used in this study. It was necessary to determine the appropriate number of preconditioned CVMSCs and the specific dose of their secretory component which had a measurable effect on the performance of cancer cells. These spatiotemporal effects were measured while treating the cancer cells directly, either with the naïve CVMSCs and their CM or with the cellular component and the secretome of the preconditioned CVMSCs, at various doses of CM or at different cellular ratios, before measuring their functional outcome.

First, we evaluated the viability of CVMSCs, which was not affected by high concentrations (up to 100%) of CM-MDA231. Although CM-MDA231 contains various pro-inflammatory as well as pro-apoptotic molecules, including interleukins and matrix metalloproteinases [40,41,42,43,44], the resistance of CVMSCs to harmful effects has already been demonstrated with their resistance and survival against detrimental effects of H_2_O_2_ and glucose [32,33].

To specify the differential effect of preconditioned CVMSCs and their secretomes on the cancer and on normal cells, our studies showed that naïve or preconditioned CVMSCs and their CM did not modify the survival of the HMECs at any of the cellular ratios tested, or with the different concentration of CM. Although treatment of MDA231 cells with the CM of CVMSCs at various concentrations for 24 H and 48 H did not change the viability of the MDA231 cells, sustained treatment for 72 H resulted in a significant decrease in cellular viability at both 10% and 25% CM concentration, as observed in Figure 1. We have earlier reported that under the influence of cancer conditioned media mimicking the cancer microenvironment, CVMSCs express a variety of anti-proliferative proteins such as IL-27, MSTN, and TGF-β2, and pro-apoptotic proteins including IFN-α2 and FASLG [23]. The expression of these molecules and their activity may be responsible for suppressing the proliferative potential of the MDA231 cells. Among them, evidence obtained in preclinical tumor models has indicated that IL-27 has a potent antitumor activity, not only through induction of tumor-specific Th1 and cytotoxic T lymphocyte (CTL) responses, but also having direct inhibitory effects on tumor cell proliferation, survival, invasiveness, and angiogenic potential [45]. Since HMECs did not create a conducive environment for CVMSCs to exert any such effect that may in turn have been detrimental for their physiological activities, no change in cellular phenotype was observed. Therefore, it is understandable that the treatment of MDA231 cells with the CM showed a significant decrease (Figure 1B(iii)) in their overall viability as compared to the HMEC cells (Figure 1A(iii)) treated with the same doses under similar conditions. It has been reported that MSCs secrete and express several essential effector molecules, including cytokines, chemokines and growth factors, etc., and thus exert their influence on the target cells, resulting in immunomodulation, tissue regeneration, and pro- and anti-apoptosis [46]. However, the selective impact of CM-CVMSCs on the cancer cells and not on the HMECs may be because of the interaction of the unidentified components present in the CM with the secretory factors of the cancer cells, that are absent in the HMEC secretome. That may eventually bring about a change in their deferred survival compared to the HMECs. However, further investigation is needed to identify those expressed, as well as the target molecules.

Similar results were observed in MDA231 cells, which showed a significant decrease in cellular viability after sustained treatment for 72 H with the cellular component of the preconditioned CVMSCs at the ratio of 1:5 (MDA231: CVMSCs) (Figure 2B(iii)). However, no such modulation was observed in HMEC cells treated with preconditioned cells at any of the time points or the cellular ratios tested (Figure 2B(i–iii)). In addition to the CM, the cellular component of MSCs use an alternative mechanism called paracrine effect, where MSCs secrete biologically active factors that exert their modulatory effects, including angiogenesis, tissue regeneration, apoptosis, inflammation, migration, and gene expression, on the target cells [47,48,49,50,51,52]. However, in vitro and in vivo studies have shown that different cell types respond differently to the paracrine signaling from MSCs, causing the modulation of many cellular responses [50]. That may explain the differential outcome of the cellular component of preconditioned CVMSCs on the MDA231 cells and the HMECs.

A decrease in the MDA231 cellular viability after treatment with secretory (Figure 1B(i,ii)) or cellular products (Figure 2B(i,ii)) of the preconditioned CVMSCs supports the concept that their sustained treatment may modulate other functional characteristics of the MDA231 cells. Suppression in cellular adhesion increases the chances of the detachment of cancer cells from their primary sites into the lymphatic or blood stream, taking them to distant sites where they settle, proliferate, and result in a new tumor, the process known as metastasis. Intercellular adhesion, or their adhesion with the extracellular matrix, plays a pivotal role in tissue architecture and integrity. It regulates cellular proliferation, cellular migration, and invasion, thereby regulating the metastasis in various cancers [53,54]. Treatment with the CM or the co-culture of preconditioned CVMSCs with MDA231 cells did not alter the adhesion potential of the MDA231 cells significantly as compared to the untreated controls (Figure 2A and Figure 3A). As reported earlier [23], in a cancer microenvironment, CVMSCs secrete pro-adhesive molecules that may be responsible for regulating the adhesive properties of MDA231 cells through their secretome or via the paracrine effect.

MDA231 cellular proliferation reduced significantly after co-culturing with preconditioned CVMSCs, as well as after treatment with their CM, as observed in Figure 2B and Figure 3B. It has been reported that umbilical cord MSCs reduce proliferation and induce apoptosis in the U251 human glioma cell line [55]. Furthermore, the co-culture of MSCs isolated from different sources suppress tumor growth and proliferation in brain [18,55,56,57,58], breast [59,60], lung [55] colorectal [61], ovarian [62], and esophageal [63] cancers. Modulation in the expression of various oncogenes and tumor suppressor genes was observed in MDA231 cells after their co-culture with preconditioned CVMSCs, as well as after treatment with their secretory products (Table 1). Important tumor suppressor genes which depicted increased expression included *CDKN1A* [64], *CDKN1C* [65], *IFN-γ* [66], *CDH1* [67], and *RB1* [68], etc., whereas the prominent downregulated oncogenes in MDA231 cells after their treatment with CVMSCs included *AKT* [69], *Cyclin D1* [70], *CSF1* [71], *EGFR* [72], *IL6* [73], *JUN* [74], and *TGF-β1* [75], etc. Modulation in the expression of these genes may play an important role in regulating the proliferation of MDA231 cells co-cultured with CVMSCs or treated with their secretome. However, the exact mechanism underlying their anti-proliferative effects on MDA231 cells will be examined in future studies.

At an advanced stage of development, cancer cells detach from the primary site and migrate to neighboring or distant tissues or organs and develop into secondary tumors [76]. Cancer cells take advantage of their migration and invasion potential to traverse the blood and lymphatic system and develop into a secondary tumor [77,78]. The process of metastasis is facilitated by a systematic process, the EMT, which is being considered a promoter of metastasis [79]. During EMT, the cells undergo various transformational changes including disruption in cell–cell adhesion, a change in cellular polarity, remodeling of the cytoskeleton, and changes in cell–matrix adhesion. It is followed by improved migratory and invasive properties [80]. The metastasis process associated with mesenchymal features is displayed in a variety of cancers, including the most aggressive breast cancer [81]. Acquiring EMT features followed by metastasis is linked to disease progression [82,83]. EMT is executed by multiple transcription factors, adhesion molecules, cytokines, and other factors. The dysregulation of these effector molecules is regarded as a driver for EMT and metastasis, followed by disease progression [84]. An analysis of transfection factors and other molecules responsible for metastasis and EMT revealed that the expression levels of a subset of genes upregulated during these transitions were decreased in MDA231 cells treated with preconditioned CVMSCs (both IC and CM) (Supplementary Table S1). These include *BMP1, COL3A1, COL5A2, FOXC2, TIMP1, VCAN, WNT5B, CALD1, CAMK2N1, CDH2, FN1, MMP9, SNAI1, SPARC,* and *TMEM132A*. Upregulation was observed in many genes, including *CAV2, FGFBP1, KRT19, MST1R, OCLN*, and *RGS2*, which play important roles in the negative regulation of EMT and metastasis phenotypes in many cancers. These genes are involved in various functions such as cell growth and proliferation, cellular migration, motility and invasion, differentiation and development, and cellular adhesion (Supplementary Table S2). However, the exact mechanisms with which the expression of these genes are modulated in CVMSCs-treated MDA231 cells is not yet known and will be examined in a future studies.

Although MDA231 treatment with the secretome of naïve or preconditioned CVMSCs did not alter their migration and invasion potential (Figure 3C,D), yet co-culture of MDA231 cells with the cellular component of preconditioned CVMSCs resulted in a significant decrease in their migration as well as invasive phenotypes (Figure 4C,D and Figure 5A,B). The difference in outcome of the co-culture setting may specifically be due to the paracrine signaling initiated by the CVMSCs in the tumor setting, where they express specific anti-migratory and anti-invasive factors, which are absent in the secretome, collected in the lab setting in the absence of the MDA231 cells. However, these factors need to be discovered and investigated. It has been previously reported that human cord blood MSCs downregulate PI3K/AKT, c-Myc/ERK and EGFR/c-Met activities, leading to a decrease in the invasion and migration potential of the glioblastoma cell line [56]. Furthermore, the involvement of Wnt signaling has also been reported to inhibit the cellular migration of breast cancer by adipose and human umbilical cord derived MSCs [85,86].

MDA231 cell proliferation decreased significantly after co-culture with preconditioned CVMSCs, as well as with their secretome. To understand the mechanism behind this outcome, we evaluated the expression status of a few cell effector molecules involved in cell proliferation and apoptosis pathways. mRNA profiling of the MDA231 cells after treatment with cellular component of CVMSCs or their secretome demonstrated modulation in the expression of multiple effector molecules that are involved in oncogenesis and tumor suppression phenotypes (Table 1).

To validate the RNA analysis data, the proteomic analysis of specific tumor suppressors and oncogenes confirmed the modulation of genes in MDA231 cells treated with the cellular component or the secretome of preconditioned CVMSCs. Flow cytometry results demonstrated a significant increase in the expression of tumor suppressor proteins CDH1 and IFN-γ, and repression in oncogenes such as IDO, IL6, MMP7, and TGF-β1, as shown in Figure 6A,B. However, which specific pathway or effector molecule precisely mediates the pro-apoptotic and anti-proliferative signals in preconditioned CVMSCs treated MDA231 cells needs to be ascertained.

The E-cadherin adhesion (CDH1) protein is important for maintaining normal tissue morphology and cellular differentiation. It acts as an invasion and metastasis suppressor protein, as its loss leads to a rapid progression into invasive and metastatic carcinomas [87]. Frequent E-Cadherin gene mutations have been found in diffuse gastric and infiltrative lobular breast carcinomas [88,89]. IFN-γ initiates both pro-tumorigenic and antitumor immunity. It acts as a cytotoxic cytokine and, with granzyme B and perforin, initiates apoptosis in tumor cells [90]. Indoleamine 2,3-dioxygenase (IDO) catalyzes the breakdown of the essential amino acid tryptophan into kynurenine, and is over-expressed in breast tumor cells and in tumor-associated cells. It has been reported that IDO expression in tumor tissues correlates with a significantly worse prognosis in patients [91]. IL-6 overexpression is associated with poor clinical prognosis and metastasis. It is frequently activated in breast cancer, and promotes metastasis while simultaneously suppressing the anti-tumor immune response [92]. MMP7 gene expression is correlated with tumor size, triple-negative (TN) status, and the recurrence of breast cancer. It is also associated with breast cancer metastasis and, importantly, metastasis to the brain and lungs [93]. TGF-β1 signaling plays a significant role in metastasis and epithelial–mesenchymal transition in breast cancer. It has also been shown to confer malignant properties, including cell motility and invasiveness, and plays critical roles in breast cancer metastasis [75].

The findings of these studies indicate that CVMSCs and their secretomes (preferentially after preconditioning) induce a significant loss of functional capabilities in the MDA231 cell line. The results suggest that CVMSCs may be considered as one of the options in the field of cellular therapy, in parallel to CAR-T cell and other similar therapies. However, these studies must be validated in animal models for efficiency, mechanism of action, safety, pharmacokinetics, and pharmacodynamics, before their application as cancer therapies in human patients.

## 4. Materials and Methods

### 4.1. Ethical Approval and Placenta Collection

The Institutional Review Board (IRB) of King Abdullah International Medical Research Centre (KAIMRC) approved this study under proposal number RC20/346/R. Informed consent was taken from expecting mothers at 38–40 weeks of gestation with uncomplicated and healthy pregnancies. All donors were admitted in the delivery rooms of King Abdulaziz Medical City for the delivery of their babies. Placenta and associated umbilical cord tissues were collected within 2–3 H of delivery of the baby. The expecting mothers were regularly monitored for the fetal age, and viability was performed during the gestational period by ultrasound examinations. Sample collection and research guidelines set by IRB were strictly followed during the clinical, laboratory and experimental procedures.

### 4.2. Reagents and Cell Lines

MDA-MB-231 (MDA231) Human Breast Cancer Cell Line (cat#HTB-26) was purchased from American Type Culture Collection (ATCC, Manassas, VA, USA). Human Mammary Epithelial Cells (HMEC; cat #A10565) were purchased from Thermo Fisher Scientific (Waltham, MA, USA). Fluorescent-labeled antibodies for flow cytometry experiments, including IFN-γ (Human IFN-gamma PE-conjugated Antibody) cat# IC285P; CDH1 (Human E-Cadherin PE-conjugated Antibody) cat# FAB18381P; IDO (Human Indoleamine 2,3-dioxygenase/IDO PE-conjugated Antibody) cat# IC6030P; IL6 (Human IL-6 PE-conjugated Antibody)cat# IC206P; MMP7 (Human MMP-7 PE-conjugated Antibody) cat# IC9071P; and TGF-β1 (Human TGF-beta 1 Alexa Fluor^®^ 488-conjugated Antibody) cat# IC10502G were purchased from R&D Systems (Minneapolis, MN, USA).

### 4.3. Isolation, Culture, and Maintenance of CVMSCs and Human Umbilical Vein Endothelial Cells (HUVECs)

CVMSCs were isolated from human term placenta using the previously described explant method [20]. Briefly, about 40 g of the chorionic villus tissue was extracted from the placenta, cleaned from other placental tissues, washed with sterile PBS, and incubated overnight at 4 °C in 2.5% trypsin (Life Technologies, NY, USA) supplemented with 270 units/mL DNase (Life Technologies, NY, USA) and antibiotics (100 U/L penicillin and 100 mg/mL streptomycin). Incubated tissue was washed with PBS and diced to smaller 1 mm explants and placed in a culture flask and left to dry for 1 H at 37 °C. Complete DMEM-F12 culture medium (Life Technologies, NY, USA) containing 10% MSC Certified Fetal Bovine Serum (Life Technologies, NY, USA), 100 mg/mL of L-glutamate, and supplemented with antibiotics, was added to the culture flask. Explants were incubated at 37 °C in a cell culture incubator containing 5% CO_2_. The culture medium was replaced every three days and the cells migrated from the explants were harvested with TrypLE^TM^ Express detachment solution (Life Technologies, NY, USA) and characterized before being used for experimental purposes. The cells were harvested at 75% confluency using TrypLE^TM^ Express detachment solution and simultaneously characterized by flow cytometry. CVMSCs at passages 3–4, prepared independently from five different placentae, were used in these studies.

HUVECs were isolated from umbilical cord vein tissues using the standard operating protocols, as described earlier [94]. The umbilical veins were washed thoroughly with PBS before digesting it with collagenase type II (cat# 17101-015, Thermo Fischer Scientific, Saudi Arabia) in PBS solution. Digested tissue was incubated for 25 min at 37 °C in a humidified incubator with 5% CO_2_ and 95% air. The liberated HUVECs were collected and resuspended in complete Endothelial Cell Growth Medium (cat# PCS-100-041™, ATCC, Manassas, VA, USA), and cultured at 37 °C in a cell culture incubator. The cells were characterized by flow cytometry before using them in the subsequent experiments.

### 4.4. Conditioned Media (CM) Collection and CVMSCs Preconditioning

CM was generated from breast cancer cell line (MDA231) cultures, using our previously reported method [20]. Briefly, 1 × 10^5^ MDA231 cells were cultured in DMEM-F12 culture medium with 10% FBS, 100 mg/mL L-glutamate and antibiotics (100 U/mL penicillin and 100 mg/mL streptomycin) until cells attained 75% confluency. Dead cells and debris were removed by washing the cell monolayer with PBS. The cells were fed with fresh complete medium and incubated further for 72 H, when conditioned medium (CM-MDA231) was collected and stored at −80 °C for future use.

CVMSC preconditioning with CM-MDA231 was performed using our previous published method [23]. Briefly, 1 × 10^5^ CVMSCs were cultured in DMEM-F12 culture medium supplemented with 10% FBS, 100 mg/mL L-glutamate, and antibiotics (100 U/mL penicillin and 100 mg/mL streptomycin) until 75% confluency. After washing the monolayer with PBS, the cells were incubated with different concentrations ranging from 5 to 25% of CM-MDA231 for 24 H and 72 H. After preconditioning, the cells were washed again and fed with complete medium and incubated for 24 H and 72 H. The conditioned medium (CM-CVMSC) was collected as described earlier [95], centrifuged to remove the dead cells and debris, and stored at −80 °C for future use. The preconditioned cells were subsequently harvested and stored for the treatment of MDA231 cells.

### 4.5. MDA231 and HMEC Treatment with CM and Preconditioned CVMSCs

For treatment with CM-CVMSCs, the CM collected from preconditioned CVMSCs was initially diluted in complete medium to a working solution of 5%, 10%, and 25% before adding to MDA231/HMECs monolayers in a six-well plate. The cells were incubated for 24 H, 48 H, and 72 H at 37 °C before performing the functional and other cell-based assays. In direct cell–cell contact (IC) experiments, the culture system consisted of CVMSCs seeded in the reverse side of a 0.4 µm pore size transwell membrane (cat# 9300402, cellQART, Northeim, Germany) and incubated for 24 H. The MDA231/HMECs were seeded in the upper chamber at a ratio of 2:1, 1:1, 1:2, and 1:5 between MDA231/HMECs and CVMSCs of the membrane in complete medium. Cells were incubated for 24, 48, and 72 H at 37 °C in a cell culture incubator before performing the functional analyses [95].

### 4.6. MTS Cell Proliferation Assay

The proliferation of MDA231 cells, untreated or treated with CM collected from preconditioned and naïve CVMSCs, and preconditioned CVMSCs (cellular component) was measured by MTS colorimetric assay kit (CellTiter 96 R Aqueous Non-Radioactive Cell Proliferation Assay, cat#G5421, Promega, Germany). MDA231 cells were treated with preconditioned CVMSCs at various cellular ratios (1:1 to 2:1 MDA231: CVMSCs) and CM at different concentrations ranging from 5% to 25%. The cells were incubated for 72 H followed by the addition of MTS by following the manufacturer’s instructions. Cells were incubated in MTS substrate for a further 4 H at 37 °C, and the color absorbance was recorded at 490 nm using a spectrophotometer plate reader (Spectra MR, Dynex Technologies, Denkendorf, Germany). Results were presented from three independent samples as mean ± standard deviation. To stop the proliferation of CVMSCs, the cells were treated with 25 µg/mL mitomycin C at 37 °C for 1 H before starting the co-culture, as previously described [95].

### 4.7. Real Time Cell Analysis (RTCA) for Cellular Functions

MDA231 cellular functions such as proliferation, migration and invasion were assessed using a xCELLigence Real-Time Cell Analyzer (RTCA-DP version; Roche Diagnostics, Mannheim, Germany) system. Cellular events were continually monitored by recording label-free changes in electrical impedance (reported as cell index), as already described [96,97,98]. For adhesion and proliferation, we used “E-Plate 16” (cat#05469813001, Roche Diagnostics, IN, USA). The wells of the plate were equilibrated with 100 µL of complete medium to set the background impedance, as previously described [99]. Each group of MDA231 cells (treated and untreated control) was seeded in four wells, followed by incubation at room temperature for 30 min to allow the cells to adhere before loading into the xCELLigence system, housed in a cell culture incubator at 37 °C. The cell index was monitored for 72 H. Cellular adhesion was measured after 2 H, and the rate of cell proliferation was calculated after 72 H. Data were analyzed using RTCA xCELLigence software (version 1.2.1), and final data for proliferation were demonstrated after normalizing them with the adhesion data. Data for adhesion and proliferation were expressed as normalized cell index with mean and standard errors.

CIM-16, a specially designed 16 well plate (cat#05665825001, Roche Diagnostics, IN, USA) was used in the xCELLigence system to record the cellular migration of untreated or CVMSCs treated MDA231 cells across the chambers of the two chambered plate. The two chambers of the CIM-16 plate, the upper and lower chambers, were separated by a porous (pore size 8 µm) polyethylene terephthalate (PET) membrane in conjunction with microelectrodes [95]. A total of 160 µL of pre-warmed complete media was added to the wells of the lower chamber and 50 µL pre-warmed serum-free media was added to wells of the upper chamber. The plates were locked in the RTCA device and incubated at 37 °C in a cell culture incubator for 1 H for equilibration, and background impedance was set as previously described [23]. Untreated or treated MDA231 cells were seeded at a density of 20 × 10^3^ in the upper chamber in 100 µL serum-free medium and the plates were incubated at room temperature for 30 min to allow the cells to adhere to the membrane. The impedance value of each well was captured by the xCELLigence system after every 15 min for 24 H, as described above. Experiments were performed with four independent samples, and the migration of cells observed in the presence or absence of 20% FBS served as positive and negative controls, respectively.

To monitor MDA231 cellular invasion after treatment with CVMSCs and its CM, HUVECs were seeded at the density of 2 × 10^4^ cells in a 16-well E-Plate to create a monolayer of cells. CVMSCs treated or untreated control MDA231 cells at a density of 1 × 10^4^ cells, were added to the HUVEC monolayer, as described earlier [95]. After 48 H, the cell invasion index (mean ± standard errors) was measured by calculating the normalized cell index at pausing time (15–20 h) of HUVEC growth. All experiments were performed with four sets of MDA231 cells treated independently with CVMSCs isolated from four different placentae.

### 4.8. In Vitro Cellular Migration and Invasion Assays

The migratory potential and invasiveness of MDA231 cells before and after treatment was assessed by performing an in vitro migration and invasion assay, as described earlier [23]. The cells were made to pass through 8-µm pore polycarbonate transwell inserts (for migration), and through a similar insert coated with Matrigel (cat#356235, BD Biosciences, San Jose, CA, USA), for determining cellular invasion. Treated and untreated MDA231 cells at a concentration of 2.5 × 10^3^ cells/mL were seeded with serum-free medium in the upper chamber of the insert. Complete medium supplemented with 20% FBS was used as a chemo-attractant and added to the lower chamber of the plate. The cells were incubated for 48 H in a humidified cell incubator in 5% CO_2_ at 37 °C. In both invasion and migration assays, the cells that had passed through the membrane to the bottom chamber were washed with PBS and fixed with paraformaldehyde for 15 min at room temperature. Staining was carried out with 0.1% crystal violet stain and the cells were visualized and photographed under a light microscope (250× magnification). In order to minimize the experimental bias, the migrated and invaded cells were counted manually under a microscope independently by three scientists (one senior and two junior scientists). The cell numbers obtained were compiled independently by each investigator, and the data files were then combined in an Excel sheet to determine the rate of migration and invasion.

### 4.9. Flow Cytometry

CVMSCs treated or untreated MDA231 cells were harvested, and 1 × 10^5^ cells were stained using fluorescent antibodies against the specific antigens, as described above in the “Reagents and Cell Lines” section and as described earlier [100]. For cell surface staining, the cells were incubated with respective antibodies for 30 min and washed with cold PBS at 4 °C. For the analysis of intracellular expression of the proteins, the cells were washed with PBS, fixed with paraformaldehyde in sterile PBS for 10 min at room temperature, and permeabilized for 5 min at room temperature in 0.1% Saponin containing PBS. Intracellular and cell-surface protein expression was assayed by BD FACS CANTO II (Becton Dickinson, NJ, USA) flow cytometer. Unstained cells and IgG or IgM isotype antibodies were used as a negative control.

### 4.10. RNA Isolation and Real-Time PCR (RT-PCR)

We used an RNEasy mini kit (cat#74104, Qiagen, MD, USA) to isolate total RNA from MDA231 cells of all experimental groups. A QuantiTect Reverse Transcription Kit (cat# 205313, Qiagen, MD, USA) was used to transcribe RNA into the single stranded cDNA. Real-time PCR reaction was performed to detect the expression of 84 genes related to Human Breast Cancer using an RT^2^ Profiler Kit (cat# PAHS-131ZA, Qiagen, Hilden, Germany) and the RT² Profiler PCR Array Human Epithelial to Mesenchymal Transition (Qiagen Cat# PAHS-090ZA) to analyze modulation in the expression of genes responsible for EMT on the CFX96 real-time PCR detection system (Bio-Rad, CA, USA). Data analysis was performed using the CFX manager software (Bio-Rad, CA, USA). The data were analyzed by calculating ΔΔ^−2^ values and expressed as fold change expression, as compared to the relative expression of GAPDH used as a loading control and the untreated MDA231 cells as an experimental control. Experiments were repeated three times using CVMSCs isolated from five different placentas.

### 4.11. Statistical Analysis

Bar graphs show the data with means ± standard error (SE) from three independently executed experiments. To avoid bias, the experiments were repeated independently. An unpaired *t*-test was used for data comparison between two groups. For the single data factor, two groups were compared using one-way analysis of variance (ANOVA), while data of double factors in multiple groups were compared by two-way ANOVA. The results were further analyzed using the Mann–Whitney tests for two group comparisons, and the Kruskal–Wallis test for more than three groups. A *p* value of ≤0.05 was considered to be statistically significant.

## 5. Conclusions

We investigated the feasibility and usability of CVMSCs and their secretory products (preferentially after preconditioning) in cellular therapy against cancer. The conditioned media collected from the preconditioned CVMSCs inhibited proliferation, but did not alter the adhesion, migration, and invasion potential of MDA231 cells. Contrarily, the cellular component of preconditioned CVMSCs restricted all the functional activities of MDA231 cells, including proliferation, migration, and invasion, although no change in adhesion was observed after treatment. Under the influence of CVMSCs and its secretome, the MDA231 cells depicted the upregulation of multiple pro-apoptotic genes and the downregulation of many oncogenes, explaining the mechanism behind their anti-tumor properties. Furthermore, the modulation of genes responsible for EMT suggests that CVMSCs minimize the invasive and metastatic phenotypes of MDA231 cells. These data demonstrate that CVMSCs and their secretory products may possibly be utilized as therapeutic agents against cancer. However, more comprehensive investigation, including the preclinical studies, pharmacokinetics, and pharmacodynamics, will further establish them as the choice of therapy for cancer patients.

## Figures and Tables

**Table 1:** Modulation in gene expression of MDA231 cells after treatment with CVMSCs: differential gene expression was observed in MDA231 cells after treatment with CM at 25% and with preconditioned CVMSCs at 1:5 ratio. The results were normalized with untreated controls and with GAPDH as an internal control. RT-PCR was performed using RT^2^ Profiler Kit™, as described in the Materials and Methods section. Three independent experiments were performed using CM and cells isolated from five placentas. Data are expressed as fold change calculated from the ΔΔ^−2^ values.

	Oncogenes			
			Fold Change Expression as Compared to Control	
	Gene Symbol	Gene Name	CVMSCs CM 25% Treatment	MDA231:CVMSCs (1:5) Treatment
1	AKT1	AKT Serine/Threonine Kinase 1	0.618622154	0.150137447
2	ATM	Ataxia Telangiectasia Mutated	1.778029701	0.000298889
3	BIRC5	Baculoviral IAP Repeat Containing 5	1.233057039	0.619744644
4	CCND1	Cyclin D1	0.290049141	0.231581661
5	CCND2	Cyclin D2	1.287981276	0.040808033
6	CCNE1	Cyclin E1	1.864428555	0.004532964
7	CSF1	Colony Stimulating Factor 1	0.348220492	0.086619766
8	EGFR	Epidermal Growth Factor Receptor	0.569847063	0.001123859
9	ERBB2	erb-b2 Receptor Tyrosine Kinase 2	0.598625231	0.00608788
10	ESR1	Estrogen Receptor Alpha	1.379391323	0.070357461
11	GRB7	Growth Factor Receptor-Bound Protein-7	1.307511163	0.737863692
12	GSTP1	Glutathione-S-transferase Pi 1	0.187847314	0.41971974
13	IDO	Indoleamine-2,3-Dioxygenase Enzyme	0.285609401	0.269798431
14	IGF1R	Insulin like Growth Factor 1 Receptor	0.588756921	0.174552115
15	IGFBP3	Insulin-like Growth Factor Binding Protein 3	1.26035563	0.80957975
16	IL6	Interleukin-6	0.759431623	0.001836595
17	JUN	Jun Proto-oncogene.	0.479301652	0.003486001
18	KRT18	Keratin 18	0.578317336	0.535014722
19	KRT8	Keratin8	1.239435925	0.279858319
20	MKI67	Marker of Proliferation Ki-67	0.538599614	0.000380797
21	MMP2	Matrix Metalloproteinase 2	1.801857196	0.007483997
22	MMP7	Matrix Metalloproteinase 7	1.562817349	0.870375475
23	MUC1	Mucin 1	0.659100118	0.431684903
24	NOTCH1	Neurogenic Locus Notch Homolog Protein 1	1.081148284	0.350957369
25	PLAU	Urokinase-Plasminogen Activator	0.428671888	0.69880195
26	PTGS2	Cyclooxygenase 2	1.156198717	0.003074883
27	SLC39A6	Solute Carrier Family 39 Member 6	1.348045319	0.819709278
28	TFF3	Trefoil Factor 3	1.163580106	0.003049453
29	TGFB1	Transforming Growth Factor	0.364712528	0.000123789
	**Tumor Suppressor Genes**			
1	CDKN1A	Cyclin-dependent Kinase Inhibitor A	1.467611692	2.072603835
2	CDKN1C	Cyclin Dependent Kinase Inhibitor 1C	2.097303084	3.08391553
3	IFN-γ	Interferon Gamma	3.946022877	6.013921647
4	CDH1	E-Cadherin	1.80114776	2.529275488
5	RB1	Retinoblastoma Protein	1.210082072	2.477135473
6	SFRP1	Secreted Frizzled Related Protein 1	29.89537164	87.56450986

## Data Availability

All the data generated in this study are included in this manuscript and will be available upon request.

## Supplementary Materials

The following supporting information can be downloaded at: https://www.mdpi.com/article/10.3390/ijms24119569/s1.
