# Supplementary material for: Preconditioned Chorionic Villus Mesenchymal Stem/Stromal Cells (CVMSCs) Minimize the Invasive Phenotypes of Breast Cancer Cell Line MDA231 In Vitro
Source: Int J Mol Sci. 2023 May 31;24(11):9569. doi: 10.3390/ijms24119569 (PMC10253955; doi:10.3390/ijms24119569)
Supplement: Supplementary file 1 [file ijms-24-09569-s001.zip › ijms-2293480-supplementary.pdf]

Gene Symbol	Gene Name	Fold change expression as compared to control ($\Delta\Delta^{-2}$)		Functions	Expression during EMT
		CVMSCs CM 25% Treatment	MDA231:CVMSCs (1:5) Treatment		
BMP1	Bone morphogenetic protein 1	0.31457861	0.423930942	Differentiation and development	Up regulated in EMT
COL3A1	Collagen, type III, alpha 1	0.210196921	0.219638524	Differentiation and development	Up regulated in EMT
COL5A2	Collagen, type V, alpha 2	0.439233158	0.339223583	Differentiation and development	Up regulated in EMT
FOXC2	Forkhead box C2 (MFH-1, mesenchyme Forkhead 1)	0.21097502	0.465788049	Differentiation and development	Up regulated in EMT
TIMP1	TIMP metallopeptidase inhibitor 1	0.457373621	0.572261707	Cell growth and proliferation	Up regulated in EMT
VCAN	Versican	0.681056889	0.631578356	Cell growth and proliferation	Up regulated in EMT
WNT5B	Wingless-type MMTV integration site family, member 5B	0.348525954	0.712447107	Differentiation and development	Up regulated in EMT
CALD1	Caldesmon 1	0.331233296	0.544223451	Cell migration & motility	Up regulated in EMT
CAMK2N1	Calcium/calmodulin-dependent protein kinase II inhibitor 1	0.642126985	0.232568332	Cell migration and invasion	Up regulated in EMT
CDH2	Cadherin 2, type 1, N-cadherin (neuronal)	0.522896702	0.42279424	Cell adhesion	Up regulated in EMT
FN1	Fibronectin 1	0.320500354	0.796630292	Cell adhesion	Up regulated in EMT
MMP2	Matrix metallopeptidase 2	0.471211476	0.540041972	Cell adhesion	Up regulated in EMT
MMP9	Matrix metallopeptidase 9	0.491001823	0.75769821	Cell adhesion	Up regulated in EMT
SNAI1	Snail homolog 1 (Drosophila)	0.397355466	0.830569829	Differentiation and development	Up regulated in EMT
SPARC	Secreted protein, acidic, cysteine-rich (Osteonectin)	0.521939658	0.466027356	Adhesion, proliferation & survival	Up regulated in EMT
TMEM132A	Transmembrane protein 132A	0.928911233	0.527031482	Cell proliferation, migration, and invasion	Up regulated in EMT
CAV2	Caveolin 2	8.321370142	6.893132084	Cell growth and proliferation	Down regulated in EMT
FGFBP1	Fibroblast growth factor binding protein 1	1.553135227	1.710278924	Cell growth and proliferation	Down regulated in EMT
KRT19	Keratin 19	27.63671516	13.72130731	Differentiation & development	Down regulated in EMT

MST1R	Macrophage stimulating 1 receptor	14.66415237	7.438187324	Differentiation & development	Down regulated in EMT
OCLN	Occludin	10.69998803	9.071285786	Cell proliferation, migration, and invasion	Down regulated in EMT
RGS2	Regulator of G-protein signaling 2, 24kDa	2.067353913	2.400613454	Cell proliferation, migration, and invasion	Down regulated in EMT
AKT1	V-akt murine thymoma viral oncogene homolog 1	0.92188321	0.972168184	Cell growth and proliferation	Up regulated in EMT
BMP7	Bone morphogenetic protein 7	0.320745456	0.394267867	Cell growth and proliferation	Up regulated in EMT
CTNNB1	Catenin (cadherin-associated protein), beta 1, 88kDa	0.924484926	0.862160076	Cell growth and proliferation	Up regulated in EMT
EGFR	Epidermal growth factor receptor	0.497969242	0.328864927	Cell growth and proliferation	Up regulated in EMT
ERBB3	V-erb-b2 erythroblastic leukemia viral oncogene homolog 3 (avian)	3.730615409	1.540392262	Cell growth and proliferation	Up regulated in EMT
F11R	F11 receptor	0.486878064	0.276545823	Cell adhesion	Up regulated in EMT
ILK	Integrin-linked kinase	0.678021783	0.471756591	Cell growth and proliferation	Up regulated in EMT
ITGB1	Integrin, beta 1	0.55020778	0.470954509	Cell migration & motility	Up regulated in EMT
NODAL	Nodal homolog (mouse)	0.673138895	0.738787	Cell migration & motility	Up regulated in EMT
PDGFRB	Platelet-derived growth factor receptor, beta polypeptide	0.3885199	0.572850994	Cell migration & motility	Up regulated in EMT
TGFB1	Transforming growth factor, beta 1	0.588894227	0.529881915	Cell adhesion	Up regulated in EMT
TGFB2	Transforming growth factor, beta 2	0.56277634	0.863204525	Cell adhesion	Up regulated in EMT
TGFB3	Transforming growth factor, beta 3	0.744627659	0.84851699	Cell adhesion	Up regulated in EMT

Supplementary Table S1. Modulation in expression of genes in MDA231 cells responsible for Epithelial to Mesenchymal Transition (EMT), after treatment with CVMSCs. Differential gene expression involved in EMT was observed in MDA231 cells after treatment with CM at 25% and with preconditioned CVMSCs at 1:5 ratio. RT-PCR assay was performed using RT² Profiler PCR Array (Human Epithelial to Mesenchymal Transition) system, as described in material and methods section. The results were normalized with untreated controls and with GAPDH as internal control. Three independent experiments were performed. Data are expressed as fold change calculated from the $\Delta\Delta^{-2}$ values.

Cell Growth & Proliferation	Cell Migration & Motility	Differentiation & Development	Extracellular Matrix (ECM) & Cell Adhesion Molecules
AKT1		AKT1	
BMP1		BMP1	BMP1
BMP7		BMP7	BMP7
		COL3A1	COL3A1
		COL5A2	COL5A2
		CTNNB1	CTNNB1
CAV2	CAV2		
EGFR (ERBB1)	EGFR (ERBB1)		EGFR (ERBB1)
ERBB3		ERBB3	ERBB3
		F11R	F11R
	FN1		FN1
FOXC2		FOXC2	FOXC2
ILK			ILK
	ITGB1		ITGB1
JAG1	JAG1	JAG1	
MST1R (RON)	MST1R (RON)	MST1R (RON)	
NODAL	NODAL	NODAL	
	RAC1		RAC1
PDGFRB	PDGFRB		
TGFB1	TGFB1		TGFB1
TGFB2		TGFB2	TGFB2
TGFB3		TGFB3	
TIMP1			TIMP1

Supplementary Table S2. List of genes with modulated expression in MDA231 cells, after treatment with CVMSCs. Significant reduction in expression of genes involved in metastasis phenotypes was observed in MDA231 cells after treatment with CM at 25% and with cellular component of preconditioned CVMSCs at 1:5 cellular ratios. These genes are involved in various functions such as, cell growth and proliferation, cellular migration, motility and invasion, differentiation and development, and cellular adhesion. Results were obtained using RT^2 Profiler PCR Array (Human Epithelial to Mesenchymal Transition) system, as described in material and methods section. The results were normalized with untreated controls and with GAPDH as internal control. Three independent experiments were performed. Data are expressed as fold change calculated from the $\Delta\Delta^{-2}$ values.

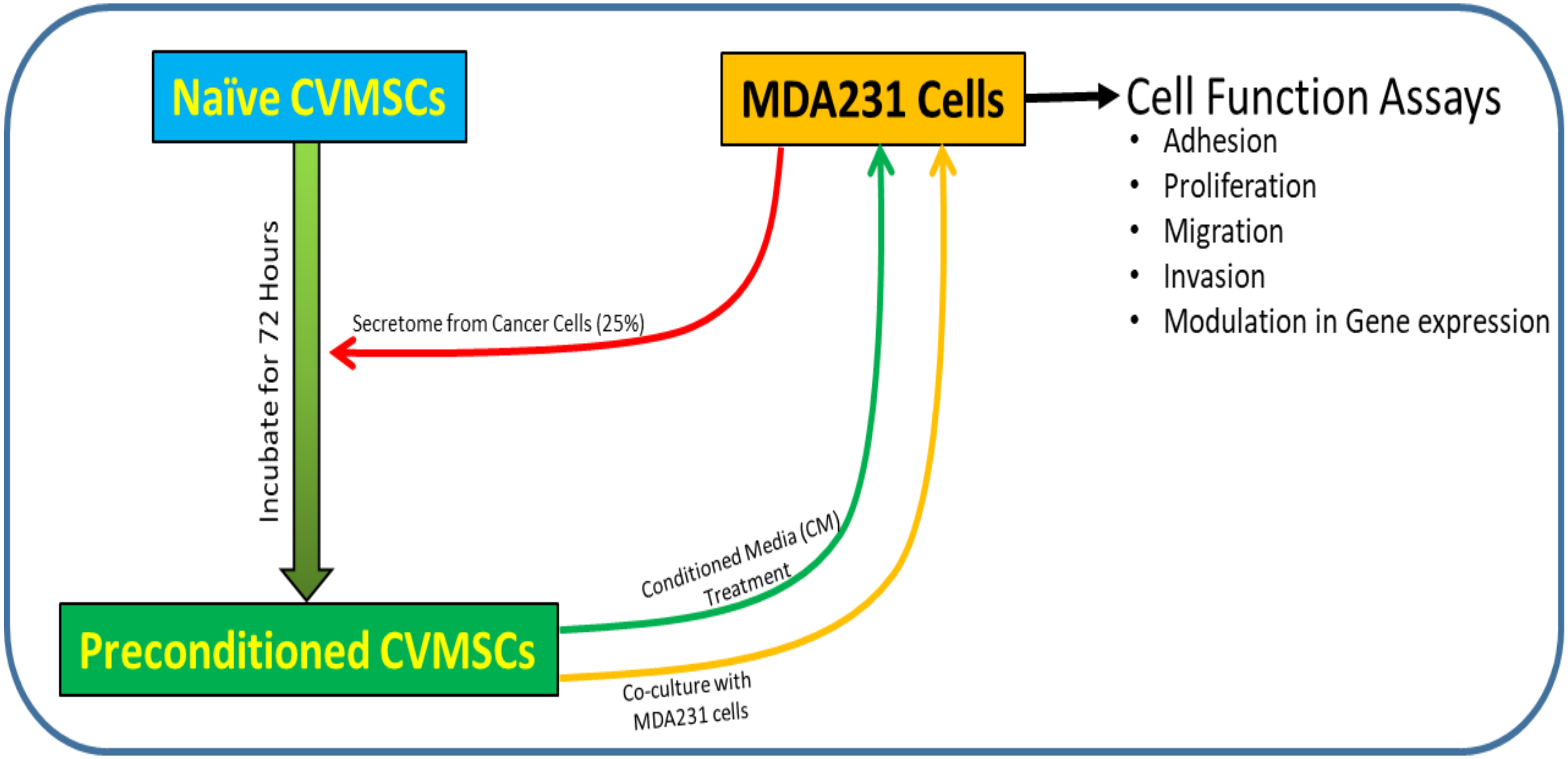

Supplementary Figure S1. **Schematic representation of the experimental plan**. Naïve CVMSCs were preconditioned by treatment with secretome (25%) isolated from MDA231 cells, for 72 hours. MDA231 cells were incubated with the conditioned medium (CM) collected from preconditioned CVMSCs, as well as with the cellular component of the preconditioned CVMSCs. After treatment (with CM) and co-culture (with cellular component), the cellular functional assays were performed on the MDA231 cells, including the cellular adhesion, cellular proliferation, migration and the invasion. Furthermore, the modulation in gene and protein expression after treatment and co-culture was assessed in MDA231 cells by mRNA analysis and flow cytometry.
